# Supplementary material for: Smooth Interpolating Curves with Local Control and Monotone Alternating Curvature
Source: Comput Graph Forum. 2022 Oct 6;41(5):25–38. doi: 10.1111/cgf.14600 (PMC9827861; doi:10.1111/cgf.14600)
Supplement: Supplementary file 1 — Supplement Material [file CGF-41-25-s001.zip › Local-Smooth-Interpolating-MonoCurvature/extern/clothoids/docs/api-cpp/variable_a00119_1a05bb4344e0f2418b6dad387247986617.html]

Variable G2lib::intersect\_with\_AABBtree — Clothoids v2.0.9

### Navigation

- index
- toc
- next
- previous
- Clothoids »
- C++ API »
- Variable G2lib::intersect\_with\_AABBtree

# Variable G2lib::intersect\_with\_AABBtree¶

- Defined in File G2lib.cc

## Variable Documentation¶

bool G2lib::intersect\_with\_AABBtree = true¶

### Quick search

### Table of Contents

- Matlab Interface Manual
- C++ API
- MATLAB API

«
hide menu

menu
sidebar
»

### Navigation

- index
- toc
- next
- previous
- Clothoids »
- C++ API »
- Variable G2lib::intersect\_with\_AABBtree

© Copyright 2021, Enrico Bertolazzi and Marco Frego.
Created using Sphinx 4.2.0.
